# Significant hearing loss in Fabry disease: Study of the Danish nationwide cohort prior to treatment

**DOI:** 10.1371/journal.pone.0225071

**Published:** 2019-12-06

**Authors:** Puriya Daniel Yazdanfard, Christoffer Valdorff Madsen, Lars Holme Nielsen, Åse Krogh Rasmussen, Jørgen Holm Petersen, Alka Seth, Søren Schwartz Sørensen, Lars Køber, Ulla Feldt-Rasmussen

**Affiliations:** 1 Department of Medical Endocrinology, Copenhagen University Hospital (Rigshospitalet), Copenhagen University, Copenhagen, Denmark; 2 Department of Otorhinolaryngology, Head and Neck Surgery and Audiology, Copenhagen University Hospital (Rigshospitalet), Copenhagen University, Copenhagen, Denmark; 3 Department of Public Health, Section of Biostatistics, University of Copenhagen, Copenhagen, Denmark; 4 Department of Radiology, Copenhagen University Hospital (Rigshospitalet), Copenhagen University, Copenhagen, Denmark; 5 Department of Nephrology, Copenhagen University Hospital (Rigshospitalet), Copenhagen University, Copenhagen, Denmark; 6 Department of Cardiology, Copenhagen University Hospital (Rigshospitalet), Copenhagen University, Copenhagen, Denmark; University of Washington, UNITED STATES

## Abstract

**Background:**

Fabry disease (FD) is a lysosomal storage disorder resulting in systemic accumulation of globotriaosylceramide resulting in multi-organ dysfunction e.g. cerebral, cardiac, renal and audiologic complications. The audiologic involvement in FD has often been neglected; while not a lethal aspect of the disease, hearing loss can have a significantly negative impact on quality of life.

**Objectives:**

To investigate baseline hearing status of the Danish Fabry cohort prior to treatment, compared to sex- and age-expected hearing levels and correlating hearing to renal and cerebral findings.

**Material and methods:**

Retrospective study of baseline hearing status of the Danish Fabry cohort (n = 83, 9–72 years). Air conduction and speech discrimination scores were assessed at 6 frequencies between 0.25–8 kHz bilaterally. Data were collected between 2001–2014 and compiled in STATA using multilinear mixed modelling for statistical evaluation.

**Results:**

Hearing thresholds at all frequencies deviated from the expected thresholds of an otologically normal cohort (p<0.001) and ranged 0.5 to 1.5 standard deviations below expected values. In total 29 males and 54 females were included. Hearing loss was more pronounced in the higher frequencies. There was a trend of association between hearing loss and measured glomerular filtration rate (mGFR) (p = 0.084). No association was present between hearing loss and albuminuria (p = 0.90), Fabry related cerebral abnormalities (p = 0.84) and cardiac left ventricular mass index, (LVMi) (p = 0.67) independent of sex. Hearing thresholds were poorer for men compared to women (p = 0.001). Sex differences were present at 0.25, 4 and 8 kHz.

**Conclusion:**

Our findings demonstrated significant hearing loss in Danish FD patients before treatment initiation, being more profound than in otologically healthy individuals at all frequencies. Additionally, we observed no association between hearing loss and LVMi, albuminuria or FD cerebral abnormalities, with a trend of association to mGFR.

**Synopsis:**

Patients with Fabrys disease have hearing loss of all frequencies and most prominently at high frequencies (4–8 kHz), with no association between the hearing loss and cerebral abnormalities, and cardiac mass but with a trend of association to measured glomerular filtration rate.

## Introduction

Anderson-Fabry disease (FD) is an X-linked lysosomal storage disorder which affects glycosphingolipid catabolism due to alfa galactosidase deficiency. This results in systemic accumulation of predominantly globotriaosylceramide (Gb3) in plasma and lysosomes, resulting in various clinical complications [1]. including but not restricted to angiokeratomas, acroparesthesia, abdominal pain, diarrhoea, neuropathy, hypohidrosis, cerebral lesions and ocular involvement [1,2]. Furthermore, severe complications include cardiac and renal manifestations ultimately resulting in cardiac and renal failure [3]. Men are typically more affected than women due to the hemizygote nature but women may present as mosaics with varying degrees of symptoms and clinical manifestations, due to at least in part skewed X chromosome inactivation [4]. FD is diagnosed by genetic analysis and confirmed biochemically by decreased alfa-galactosidase activity below the lower reference limit and accumulation of GB3/lyso-GB3 in serum and urine.

The otologic involvement in FD has often been neglected and while not a lethal aspect of the disease, hearing loss can have a significant impact on quality of life. Few studies have shown sensorineural hearing loss in Fabry patients. Hearing loss was found in 16.7% of the women and 46.7% of the men in 37 Japanese Fabry patients [5]. Likewise, the Dutch cohort comprised 97 Fabry patients, 16.8% of whom showed some degree of hearing impairement prior to treatment, both for men and women [6].

The present study aimed to determine the hearing status of the Danish Fabry cohort in 83 patients prior to treatment. The assessments were corrected in accordance to the expected age and sex based hearing decline. Finally, the association between hearing loss and renal, cardiac and cerebral abnormalities as markers of severity of disease was studied.

## Materials and methods

### 2.1 Study design and population

The study was a retrospective cross sectional study of prospectively collected data from the Danish Fabry cohort. The cohort consists of all Danish Fabry probands and their family members found by meticulous family screening since 2001. All patients independent of sex and treatment have been monitored at the University hospital of Copenhagen, Department of Endocrinology in collaboration with other specialist departments in the Fabry Team. Patients diagnosed with FD who had an audiogram prior to treatment were included (n = 83). The population has previously been partly described in other studies [7–12].

Diagnostic criteria included a Fabry positive genotype with or without a reduced alfa galactosidase activity below the lower limit of the reference interval. Based on these criteria the full cohorte comprised 88 patients. All blood related family members were offered genetic testing after a positive diagnosis was confirmed. Audiograms were collected from almost all Fabry diagnosed patients prior to treatment from 2001 to 2014 (n = 83). The most recent audiogram prior to initiation of enzyme replacement therapy was included. If the patients did not receive treatment the most recent audiogram was used.

The study was approved by the Danish Health and Medicine Authority (3-3013-667/1/), the Regional Health Research Ethics Committee (H-3-2014-FSP8) and the Danish Data Protection Agency (2014-641-0055).

### 2.2 Definitions

Pure tone average (PTA) is defined as the mean value of hearing thresholds in decibels (dB) hearing level (HL) of multiple frequencies. PTA_3_ is defined as the mean hearing thresholds in dB of 0.5, 1 and 2 kHz. PTA_4_ is defined as the mean value of hearing thresholds in dB of 0.5, 1, 2 and 4 kHz. PTA_6_ is defined as the mean hearing thresholds of all 6 frequencies (0.25, 0.5, 1, 2, 4 and 8 kHz). High frequency PTA_4,8_ is defined as the mean hearing thresholds in dB of 4 and 8 kHz. Z-PTA is the equivalent but with Z-scores instead of dB.

An otologically normal person is defined as a person in a normal state of health who at the time of testing is free from excess wax in the ear canals, is without known ear pathology and who has no history of undue exposure to noise.

### 2.3 Audiometric data

All tests were carried out by pure tone audiometry in both ears of all patients at a frequency span of 250 Hz to 8 kHz. When air conduction values were poorer than 20 dB bone conduction was also determined. Pure-tone audiometry at frequencies 0.25, 0.5, 1, 2, 4 and 8 kHz was carried out in accordance with ISO 8253–1. The modified Hughson-Westlake technique (-10/+5 dB) was employed using a Madsen Astera Audiometer, Madsen Orbiter OB 922 Clinical Audiometer and Interacoustics AC40 Clinical Audiometer, with Sennheiser HDA 200 circumaural earphones. The equipment was calibrated in accordance with IEC 60318–2, ISO 389–5, and ISO 389–8 using a Brüel and Kjaer 2610 measuring amplifier with a 4144 microphone in a 4152 coupler [13–16]. The absence of excess ear wax was secured through otoscopy. Tympanometry was not carried out on a regular basis.

Reports on hearing impairment were given based on WHO grading on hearing impairment [17]. According to WHO hearing impairment is defined based on PTA_4_ of the better ear [17]. Asymmetric hearing loss was defined as a difference between the two ears of more than 15 dB in PTA_4_. In addition, speech discrimination score was determined by presenting 25 words from a nationally validated prerecorded list contained on a CD. Each ear was assessed individually through the same headset used for pure tone measurements. The score was calculated as the percentage of words the patient could successfully report back to the analyst after each presented word. Speech discrimination scores above 90% were considered normal. All patients could speak Danish.

### 2.4 Other clinical variables

Measured glomerular filtration rate (mGFR) values collected within one month of the auditory assessments were included (n = 75). The method for mGFR measurements has been previously described [11].

24-h urine samples were collected by patients at home, the last 24 h before hospital visit. Urine samples were analysed for total albumin content based on the total urine volume and albumin concentration.

Transthoracic echocardiographies were performed using a Philips IE 33. Left ventricular (LV) wall thickness and chamber dimensions were measured by two dimensional parasternal images. LV mass (LVM) was calculated by the American Society of Echocardiography equation and indexed to body surface area (LVMi). [9]

All cerebral magnetic resonance imaging (MRI) scans performed within six months of the auditory assesment were included (n = 59). The method for MRI has previously been described [10]. All MRI scans were assessed by a radiologist (AS) and considered abnormal if Fabry abnormalities were present [18].

### 2.5 Statistical analysis and calculations

Hearing thresholds were standardised as Z-scores calculated based on hearing thresholds of otologically normal controls presented in ISO-7029 [19]. mGFR was likewise standardised to Z-scores to correct for the expected loss in GFR as a function of age using a healthy reference population as control described in Grewal & Blake 2005 [11,20,21]. Z-scores as markers of hearing loss were compared to Z-scores for mGFR.

The statistical analyses were performed by multilinear mixed model with Z-scores as dependent variable including independent factors such as left or right ear, sex, frequencies, mGFR, albuminuria,LVMi, A-gal, Gb3 and relevant interactions. Different models were tested by likelihood-ratio tests to find the best model. Where appropiate a t-test was used to detect a difference between means. Results were considered statistically significant at the 95% confidence interval and at p-values less than 5% (P < 0.05). Data has been processed and analysed using STATA 13.0.

## Results

### 3.1 Study population and hearing thresholds

**Fig 1:**
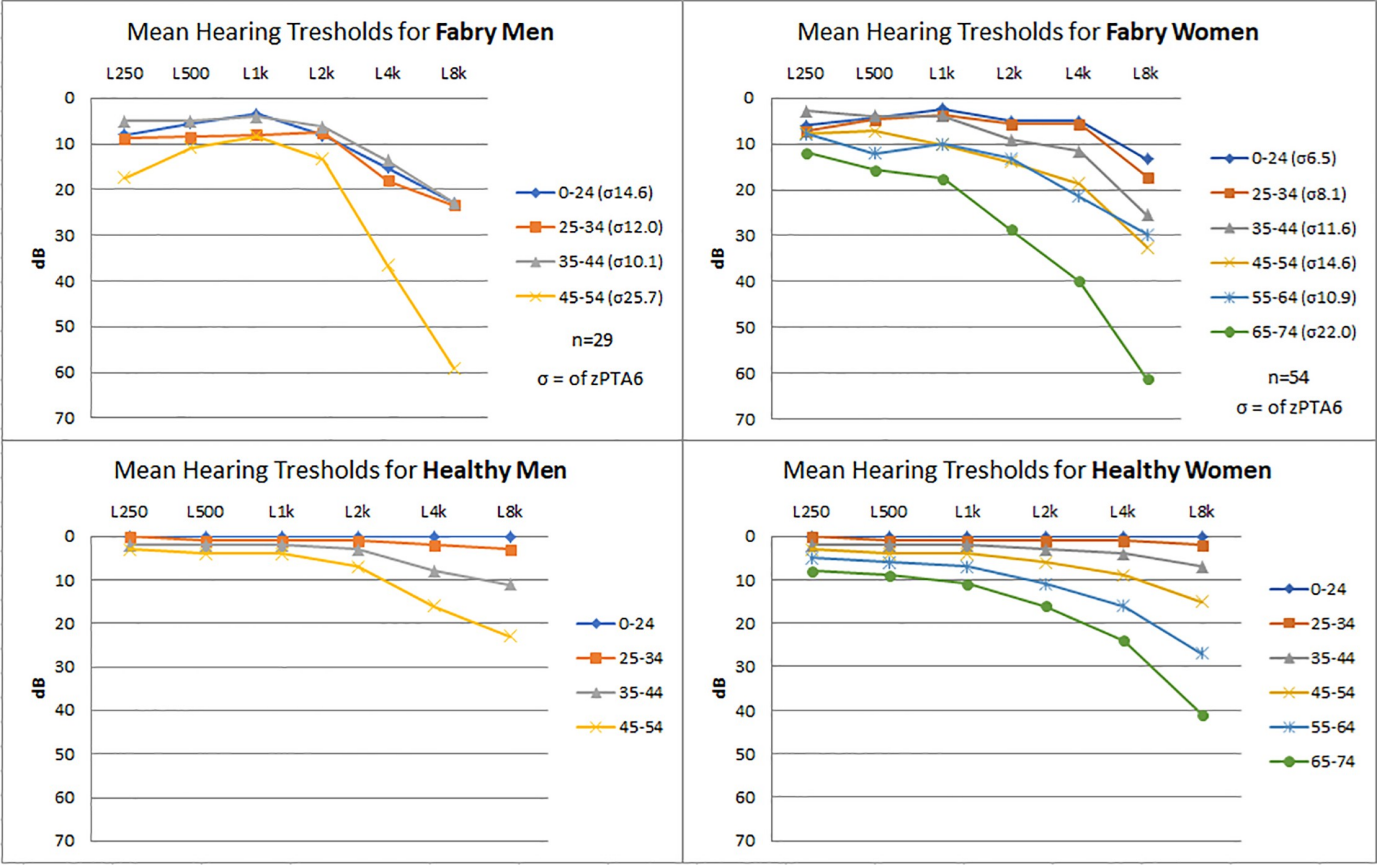
Average hearing thresholds of Fabry Patients and healthy individuals, based on age and sex. The top two diagrams show the mean hearing thresholds of both ears in dB for Fabry patients at frequencies from 0.25 kHz-8 kHz. Patients are arranged in groupes of 10 year intervals according to age. The bottom two graphs illustrate the expected hearing decay in healthy individuals based on ISO 7029. Left graphs for men and right graphs for women. σ = Standard deviations of all frequencies for the given age range (zPTA6). Hz = Hertz. dB HL = Decibels hearing level.

**Fig 2:**
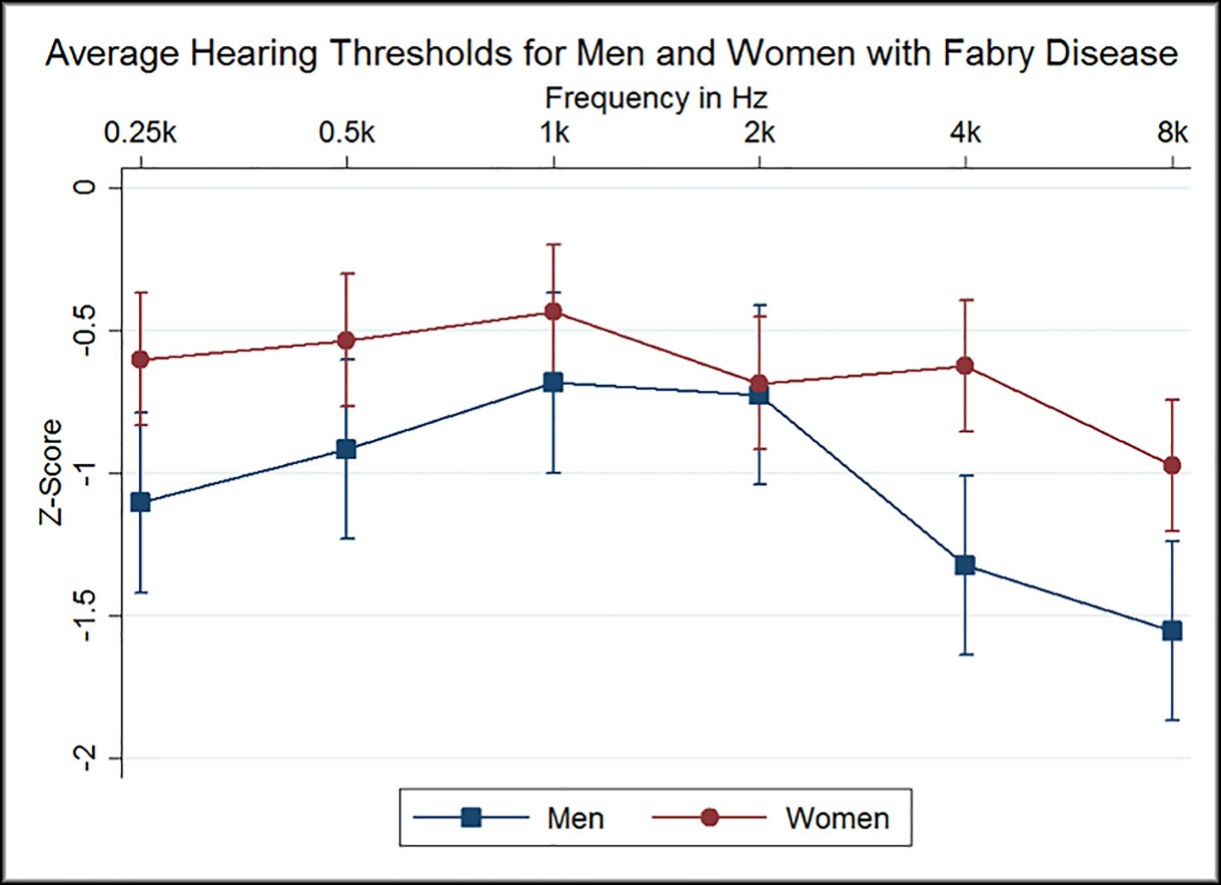
Average Hearing Thresholds for Men and Women with Fabry Disease. Mean Z-Score values of hearing levels for all Fabry patients on measured frequencies 0.25kHz-8kHz. Squares represent men and circles represent women. Confidence interval at 95%. Z-Scores indicate how many standard deviations a Fabry patients hearing threshold deviates from a sex and age corresponding healthy individual. Hz = Hertz.

A total of 83 patients, 29 males and 54 females with audiograms were included. The median age was 35 years (range 9–72 years, interquartile range 21–47), with predominantly females in the upper age groups. One male patient used hearing aids at baseline. All but one male and 41 out of 54 females were of the classic phenotype. Demographic values and general clinical findings are presented in Table 1. Genotypes and phenotypes can be seen in Table 2. Initially patients were grouped in 10 year intervals and their hearing thresholds are presented in Fig 1 as mean left and right hearing thresholds for men and women. These were compared to the expected threshold described in ISO-7029 [19] seen in Fig 1 and have subsequently been corrected by the biologically expected hearing loss depending on sex and age in Fig 2. The age and sex corrected results are presented as Z-scores.

**Table 1:** Demographic, renal and audiometric data of Fabry disease patients.

	Men (n = 29)	Women (n = 54)
**Mean age in years**	29 (9–53)	39 (10–72)
**Mean average PTA(_0.5,1,2,4 kHz_) dB HL**	9.9 (0.6–33.8)	9.4 (-0.6–44.3)
**Mean average PTA(_4,8 kHz_) dB HL**	23.2 (2.5–86.3)	20 (-1.3–71.3)
**Mean Speech Discrimination Score in %**	98.9 (68–100)	99.7 (95–100)
**Slight Impairment (26–40 dB) better ear**	1 (age 53)	0
**Moderate Impairment (41–60 dB) better ear**	0	1 (age 66)
**Asymmetric hearing >15dB**	0	0
**Mean Z-score PTA(_0.5,1,2,4 kHz_)**	-0.92 (0.16–3.31)	-0.45 (0.73–1.48)
**Mean Z-score PTA(_4,8 kHz_)**	-1.44 (0.34–6.66)	-0.59 (0–76–1.57)
**mGFR (ml/min/1.73m^2^)**	83 (21–129)^x^	92 (27–120)^†^
**Z-score mGFR/1.73m^2^**	-1.40 (2.06 - -5.70)^x^	-0.39 (0.95 - -4.47)^†^
**Albuminuria (mg/day)**	681 (4–2317)^♥^	255 (4–3284)^Ω^
**Left Ventricular Mass Index (g/m^2^)**	119 (73–271)^**μ**^	113 (50–231)^**α**^
**Globotriaosylceramide (Gb3) (μmol/L)**	7.0 (2.8–11.9)^**β**^	4.0 (2.0–8.7)^**π**^
**Alfa-Galactosidase (nmol/h/mg protein)**	1.95 (1.1–3.1) ^**β**^	15.9 (1.8–39)^**•**^

**Mean** Z-Scores indicate how many standard deviations Fabry patients parameter (Hearing threshold or mGFR) deviates from the mean of a sex and age corresponding healthy cohort.

PTA = Pure tone average (mean values of hearing thresholds), mGFR = Measured Glomerular Filtration Rate, HL = Hearing Level, Parenthesis indicate range unless otherwise stated

^x^n = **25**

^†^n = **50**

^♥^n = **16**

^Ω^n = **37**

^μ^n = **40**

^α^n = **37**

^β^n = **20**

^π^n = **44**

^•^n = **35**

**Table 2:** Genotypes and phenotypes of the Danish Fabry disease patient population.

Location	Population (n = 83)	Type of Mutation	Phenotype
**G85N**	23	Missense	CLASSIC
**R112C**	13	Missense	CLASSIC
**A156T**	12	Missense	CLASSIC
**I232T**	8	Missense	NON CLASSIC
**N34S**	7	Missense	CLASSIC
**G271S**	4	Missense	CLASSIC
**G10694**	3	Deletion	GVUS
**R342X**	3	Nonsense	CLASSIC
**R227X**	2	Nonsense	CLASSIC
**N355K**	2	Missense	CLASSIC
**G171S**	2	Missense	GVUS
**Q279R**	1	Missense	CLASSIC
**C.369+3_C.547**	1		GVUS
**R301X**	1	Nonsense	CLASSIC
**I317T**	1	Missense	CLASSIC

GVUS = Genetic variance of unknown significance

### 3.2 Individual patient hearing loss

When using WHO’s definition only one male exhibited slight hearing impairment (PTA_4_ 33.8 dB HL) and one female had moderate hearing impairment (PTA_4_ 44 dB HL) (total 2.4%). (Table 1). However, when considering the average (mean) PTA_4_ or PTA_4_ of the more affected ear three males (Mean PTA_4_ 29.5 dB HL) and two females (Mean PTA_4_ 27 dB HL) exhibited mild hearing loss (total 6%). No patient had unilateral hearing impairment. The mean high frequency PTA_4,8 kHz_ was 23.2 dB HL for men and 20.0 dB for women.

Mean speech discrimination scores were 98.9% for men and 99.7% for women. All patients, but one, had normal discrimination scores of both ears. The one male patient had a score of 68% of the left ear. This patient also exhibited a distinct hearing loss with a PTA_4_ of the left ear at 40 dB HL and a high frequency hearing threshold at 8 kHz of 95 dB HL.

None of the subjects with hearing loss exhibited air-bone gap when comparing the thresholds for air conduction and bone conduction. Thus, any hearing loses found may be categorised as sensori-neural i.e. cochlear or retrocochlear.

### 3.3 Statistical analysis with age and sex correction

Hearing thresholds at all frequencies (250Hz-8kHz) deviated significantly from the expected thresholds of a otologically healthy cohort described in ISO 7029 (p<0.001). Thus, the Fabry cohort exhibited more pronounced hearing loss in comparison to healthy age-matched individuals. Mean Z-scores for men and women ranged from 0.5 to 1.5 standard deviation scores below the population mean (Fig 2). There was no difference in hearing levels at zPTA_6_ between classic and non-classic Fabry patients (p = 0.69).

No significant difference was present between the hearing thresholds of the left and right ear in men (p = 0.37 coeff: -0.08 CI95: -0.25–0.09), and women (p = 0.33 coeff: 0.06 CI95: -0.06–0.19), respectively. Due to the absence of asymmetric hearing, results are given based on the mean hearing thresholds for the left and right ears.

### 3.4 Renal function and hearing loss

Baseline mGFR values included 75 observations and albuminuria observations totalled 53. There was a trend of association between Z-scores for hearing thresholds and renal function (p = 0.084), this association was independent of sex (p = 0.71) and being classic or non-classic (p = 0.83). There was no association between albuminuria and Z-scores for hearing thresholds (p = 0.90).

### 3.5 Cardiac left ventricular hypertrophy and hearing loss

Baseline left ventricular mass index totalled 57 measurements. There was no association between LVMi and hearing levels (p = 0.67), independent of sex (p = 0.16).

### 3.6 Cerebral MRI and hearing loss

A total of 59 patients had baseline MRI scans, and 15 (25%) had Fabry related abnormalities. There was no association between Fabry related cerebral abnomalities and hearing loss (p = 0.30), independent of sex (p = 0.16).

### 3.7 Gb3, A-gal and hearing loss

A total of 64 plasma Gb3 and 55 A-gal measurements were included. There was no association between plasma Gb3 and hearing levels (p = 0.15), this was indepedant from the patients sex (p = 0.5). There was no relationship between A-gal activity and hearing levels (p = 0.12), this was independent of sex (p = 0.48).

### 3.8 Sex based analysis

Hearing thresholds at different frequencies of Fabry patients were significantly different between men and women (p = 0.001). The variations in each individual frequency among men and women have been listed in Table 3. Statistically significant sex differences were present at 0.25, 4 and 8 kHz, with women having an average hearing advantage of 0.59 standard deviation scores (Z-score) of the mentioned frequencies. Disregarding sex differences demonstrated an average of 0.4 Z-score difference in all frequencies between men and women, however modeling with sex difference was more accurate (p = 0.007)

**Table 3:** Hearing difference between men and women with Fabry disease according to frequency.

**Frequency**	**P-value**	**Coefficient (Z-score)**	**CI 95% (Z-score)**
**♂250Hz–♀250Hz**	0.012	0.50	0.11–0.90
**♂500Hz–♀500Hz**	0.055	0.38	-0.01–0.77
**♂1kHz–♀1kHz**	0.209	0.25	-0.14–0.64
**♂2kHz–♀2kHz**	0.832	0.04	-0.35–0.43
**♂4kHz–♀4kHz**	0.000	0.70	0.31–1.09
**♂8kHz–♀8kHz**	0.004	0.58	0.19–0.97

Z-Scores indicate how many standard deviations a Fabry patient’s hearing threshold deviates from the mean of a sex and age corresponding otologically normal cohort.

♂ = male ♀ = female

## Discussion

Our findings have demonstrated that FD patients had poorer hearing compared to age-matched otologically normal individuals. Hearing loss was most prominent at high frequencies (4, 8 kHz) but also noticeable at the lowest frequency in males (250Hz). Our findings of all frequency hearing loss was partially in concert with previous studies [5,6,22,23].

When Sakurai and colleagues [5] compared hearing loss to the WHO criteria from 2003 (PTA_4_) three men (27%) and one woman (8%) had mild to profound hearing loss of the more affected ear. Likewise, Germain and colleagues concluded, in accordance to WHO 1980 guidelines, that hearing loss by PTA_3_ was present in 11 of 22 males [22], and that the majority of patients (6 out of 7) beyond 41 years of age had mild to severe hearing loss. In contrast, our cohort had only a single male older than the age of 41 (1 out of 4) with mild hearing loss. In FD children, Suntjens and colleagues [24] demonstrated baseline sex independent hearing loss both at PTA_4_ of 6.7 dB deviation from the expected and at high frequencies with a mean deviation of 10.0 dB. This indicates that hearing impairment may begin as an early symptom in FD.

Interestingly, a recent study by Rodrigues and colleagues [25] demonstrated hearing loss at all frequencies in FD patients, indiscriminate of sex in disagreement with the present study. Only one other study by Suntjens and colleagues [6] investigated hearing prior to treatment and found hearing loss at all frequencies at baseline when compared to an otologically healthy control group.

In the present study hearing loss was classified by the recent guidelines from WHO 2013 [17], which differs from the 1980 guidelines [26]. Most importantly the 1980 guidelines based hearing loss on PTA_3_ while the 2013 guidelines based hearing loss on PTA_4_. Using PTA_3_ for depicting hearing loss may ultimately result in underreporting hearing loss in FD patients as the majority of available studies as of this date point towards high frequency hearing loss as the largest contributor to hearing loss in Fabry. Using PTA_4_ as recommended by newer guidelines may yield higher reportage of hearing loss, but still lacks the lowest and highest commonly measured frequency (0.25 and 8kHz). It may therefore be favorable to use a full PTA_6_ as a mean to report hearing loss in FD, for a more cohesive and clinically inline reportage. No internationally recognised guidelines are currently available to categorise hearing loss from high frequency thresholds.

In relation to renal function a study from Köping and colleagues [23] found an association between KDIGO categories G1-4 (eGFR) and hearing loss, when compared with the most affected ear. Germain and colleagues [22] presented a relationship between hearing loss and mGFR by observing that 8/10 men who had mGFR of <40ml/min/1.73m^2^, also had mean bilateral PTA_3_ of >25dB. However, it is worth noting that hearing thresholds were not age and sex corrected in either of these GFR comparisons. In our analysis we found no statistically significant relationship between Z-scores for mGFR and Z-scores for hearing thresholds at all frequencies in PTA_3_, PTA_4_ and PTA_6_, in keeping with two studies [5,25]. Yet, in the most recent study [25] an association between albuminuria and hearing loss was detected in contrast to our findings. Despite our finding with regards to mGFR not being statistically significant (p = 0.084) our data shows a trend of association between mGFR and hearing levels. Interestingly this did not depend on the sex of the patients nor being classic or non-classic, where we would have expected males and classic variants to have a stronger association between hearing loss and other organ involvement.

Moreover, Köping et. al. [23] described an association between hearing loss and NYHA (New Your Heart Association) classes. In our study we saw no association between LVMi and hearing levels, in keeping with two studies were LVH and hearing loss were investigated [22,25]. Additionally, we saw no relationship between A-gal activity, plasma Gb3 levels and hearing levels. It is well established that plasma Gb3 levels do not correlate well with the clinical outcome of FD [27], thus monitoring Gb3 levels for hearing loss may be of little interest. However, we had no results from lyso-Gb3 measurements, which were not available from 2001. It may be more relevant to use lyso-Gb3 levels for future comparisons.

Only one study has previously shown an association between cerebral white matter lesions (WML) and hearing loss, this is however not a baseline study [28]. The authors demonstrated that the load of WML correlated to hearing loss in male patients. In our study we demonstrated no association between hearing thresholds and the presence of any degree of FD cerebral findings. However, we did not grade the cerebral lesions—the presence of any white matter lesion was considered abnormal. While our finding suggested that hearing loss may be derived from Fabry microvasculopathy within the ear, we hesitated to conclude this, due to the limited number of our cohort. It may be more relevant to regionally asses leucoencephalopathies and other cerebral pathologies, in relation to the auditory cortex with the addition of scoring the severity of white matter lesions.

The histopathological cause of hearing loss is not quite clear. However, based on the literature no study has found any noticeable air-bone gap in hearing thresholds [22,23]. This corresponds to a sensorineural mechanism rather than a conduction issue of the middle ear. Only one human study from 1989 looked at the histopathology of the inner ear [29]. The authors performed an autopsy of the temporal bone of two Fabry patients who had bilateral, moderate sensorineural hearing loss sloping towards the high frequency region. In the organ of Corti they described atrophy of the Stria vascularis and the spiral ligament. Additionally, they found loss of outer hair cells in two of the four specimens. The authors concluded that the dysfunction is a result of the direct and indirect accumulation of Gb3.

No cure exists for hearing loss in FD patients, however hearing aids may be a useful help for patients with normal speech discrimination scores. In addition, cochlear implants were successfully applied in two male Fabry patients with sensorineural hearing loss [30]. A significant improvement of the hearing in noise was described in both patients. Thus, cochlear implantation may also be an option for selected Fabry patients who experience profound discrimination problems in noisy environments.

### 4.1 Strengths and limitations

Our data showed less hearing impairment in the FD cohort than seen in other studies. There is no direct explanation for this, but notably the Danish cohort is one of the largest single center cohorts with a near 100% complete family screening. Moreover, all genetically verified FD family members were offered full organ assessment independent of sex or symptoms, why the cohort comprised a higher proportion of females than in other studies, many of them.with few or even no organ manifestations. In addition, 14% of the patients had genetic variations of unknown significance or were of the non-classic variant, further adding to the variability. The Lack of lyso-Gb3 is a limitation, however given this is a baseline investigation most data is from prior the era of lyso-Gb3 measurements.

One of the strengths of this study is the value of using Z-scores which standardise data according to age and sex. This is particularly important when studying small groups of different sex and highly varried ages in order to subtract the naturally expected functional decay. This can, however, also be a limitation as it relies highly on accurate standards to produce correct Z-scores. Moreover, this study presents pre-treatment data, which reduced confounding factors when observing the disease itself. Furthermore, with only 83 patients there is a high risk of a type 2 statistical error, which is, however, a given limitation when studying rare disorders. Nevertheless, this is one of the largest studies to date of hearing impairment in FD.

## Conclusion

Our findings have demonstrated that hearing loss is present in the Danish FD patients and is more profound than in healthy individuals on all measured frequencies. We also demonstrated that hearing loss is most prominent at high frequencies (4, 8 kHz), and that the average Fabry male has more pronounced hearing loss than the females. There was a trend of association between hearing levels and mGFR. There was no association between hearing loss and cardiac left ventricular mass index, ambuminuria, Gb3, A-gal and cerebral abnormalities. More studies of the hearing loss in Fabry patients as well as effect of therapy are needed, preferably by multicenter investigations in order to achieve more statistical power and more reliable results.

## Supporting information

S1 File Data. Data used in the analysis.
(XLSX)
